# Navigating the ethical and legal landscape of artificial intelligence in dentistry

**DOI:** 10.34172/japid.026.4323

**Published:** 2026-04-19

**Authors:** Abdolhassan Kazemi, Adileh Shirmohammadi, Sana Kazemi, Sina Ghertasi Oskouei

**Affiliations:** ^1^Medical Philosophy and History Research Center, Tabriz University of Medical Sciences, Tabriz, Iran; ^2^Dental and Periodontal Research Center, Tabriz University of Medical Sciences, Tabriz, Iran; ^3^Department of Periodontics, Faculty of Dentistry, Tabriz University of Medical Sciences, Tabriz, Iran; ^4^Student Research Center, Urumia University of Medical Sciences, Urumia, Iran; ^5^Section of Digital Dentistry, Faculty of Dentistry, Tabriz University of Medical Sciences, Tabriz, Iran

 The rapid integration of artificial intelligence (AI) has initiated a paradigm shift in dentistry, offering unprecedented capabilities in prevention, diagnostics, treatment planning, and patient management.^1-4^ By leveraging machine learning algorithms, clinicians can now utilize advanced image analysis for disease diagnosis,^5-7^ predictive analytics for identifying high-risk patients,^8^ and sophisticated systems for personalized medicine.^9^ While these innovations hold immense promise for enhancing oral health outcomes and streamlining clinical workflows, they simultaneously introduce a complex web of ethical and legal challenges that the dental profession must urgently address.

 At the forefront of these challenges are critical ethical considerations regarding patient autonomy, informed consent, and data privacy.^10-12^ The deployment of AI-driven tools necessitates that patients fully comprehend the benefits and limitations of these systems, particularly when making informed decisions about their care. Furthermore, training robust AI models requires the collection and analysis of vast amounts of sensitive patient data. Safeguarding this information through stringent adherence to data protection regulations, such as the General Data Protection Regulation (GDPR) and the Health Insurance Portability and Accountability Act (HIPAA), is paramount.^12^ Compounding these privacy concerns is the risk of algorithmic bias; if AI systems are trained on non-representative datasets, they risk perpetuating existing healthcare disparities and delivering unequal treatment outcomes.^13,14^ Mitigating this requires prioritizing diverse data and demanding transparency to overcome the “black box” nature of complex algorithms, thereby preserving trust between the practitioner and the patient.^13,14^

 Beyond ethics, the integration of AI in dentistry introduces significant legal and regulatory complexities. As diagnostic and treatment systems become increasingly autonomous, defining liability in the event of a misdiagnosis or adverse outcome becomes challenging.^15,16^ Current legal frameworks must evolve to establish clear accountability and ensure patients have adequate recourse. Additionally, the development of proprietary AI algorithms and the AI-assisted discovery of new pharmacological or biomaterials raise novel questions regarding intellectual property rights and patentability.^16,17^ A comprehensive regulatory framework is essential—one that not only ensures data quality, integrity, and anonymization but also provides clear compliance guidelines for medical devices and data protection.^12^

 Realizing the full potential of AI in clinical practice requires a foundation built on education and collaborative policymaking. The transition from utilizing AI for sophisticated diagnostic software to its seamless integration into daily practice cannot occur in a vacuum. It demands a systemic overhaul of dental education. Ethical and practical AI training must be embedded within dental school curricula, continuing medical education, and specialized training programs.^1-4^

 Ultimately, successfully navigating the AI revolution in dentistry requires a multidisciplinary approach. By fostering collaboration among dental professionals, technologists, ethicists, and legal experts, the field can establish robust guidelines that promote transparency and accountability.^18^ Embracing these technologies with a steadfast commitment to patient-centered care will ensure that the future of dental medicine is not only technologically advanced but also ethically sound and legally secure.

## Competing Interests

 At the time of submission and publication, Dr. Shirmohammadi served as the Editor-in-Chief, and Dr. Oskouei served as an Associate Editor of the *Journal of Advanced Periodontology & Implant Dentistry* (JAPID). The authors declare no other competing interests.
